# Myopic Shift Following Clear Lens Extraction in a Patient With Plateau Iris Syndrome Reversed by Synechiolysis: A Case Report

**DOI:** 10.7759/cureus.110886

**Published:** 2026-06-15

**Authors:** Yin Yen Mun, Teck Chee Cheng, Chenshen Lam, Mae-Lynn Catherine Bastion

**Affiliations:** 1 Department of Ophthalmology, Faculty of Medicine, Universiti Kebangsaan Malaysia, Kuala Lumpur, MYS; 2 Department of Ophthalmology, Hospital Canselor Tuanku Muhriz UKM, Kuala Lumpur, MYS

**Keywords:** effective lens position, intraocular lens displacement, myopic shift, plateau iris syndrome, posterior synechiae, synechiolysis

## Abstract

This case report describes a rare mechanical etiology of postoperative refractive surprise - anterior displacement of an intraocular lens (IOL) secondary to localized posterior synechiae - in a patient with plateau iris syndrome (PIS) following combined clear lens extraction (CLE) and minimally invasive glaucoma surgery (MIGS).

A 29-year-old man with PIS and medically refractory chronic angle-closure glaucoma underwent an uneventful left-eye CLE, goniosynechiolysis, implantation of a toric extended depth-of-focus (EDOF) IOL, and insertion of three iStent infinite® (Glaukos Corporation, Aliso Viejo, CA, USA) devices. Five weeks postoperatively, he presented with blurred vision, asthenopia, and binocular diplopia associated with a sudden -2.50 D myopic surprise. Dynamic slit-lamp examination revealed localized posterior synechiae extending from 1 to 5 o’clock. These adhesions exerted asymmetrical traction on the capsular bag, resulting in anterior displacement and temporal tilt of the IOL complex without pupillary block. The patient subsequently underwent targeted synechiolysis under local anesthesia. Postoperative biometry demonstrated an increase in anterior chamber depth (ACD) from 2.90 mm to 3.02 mm, confirming posterior repositioning of the IOL. Restoration of the effective lens position (ELP) reversed the myopic shift and resolved the patient’s symptoms.

This case highlights localized posterior synechiae as a rare but reversible cause of postoperative refractive surprise and underscores the importance of meticulous dynamic slit-lamp evaluation and ACD monitoring in patients with complex anterior segment conditions presenting with unexpected refractive outcomes. Timely synechiolysis may serve as a definitive and restorative intervention.

## Introduction

Plateau iris syndrome (PIS) is characterized by persistent angle closure due to an anteriorly positioned ciliary body, which persists despite a patent laser peripheral iridotomy (LPI). While argon laser peripheral iridoplasty (ALPI) serves as a standard next-line intervention, refractory cases often necessitate surgical management, including lens extraction, goniosynechiolysis, glaucoma filtering surgeries, or cyclodestructive procedures [1].

Clear lens extraction (CLE) with intraocular lens (IOL) implantation has become an increasingly popular strategy for widening the anterior chamber angle in patients with PIS [2]. In these procedures, particularly with premium IOLs, postoperative refractive outcomes are highly dependent on the effective lens position (ELP). Even minor variations in the axial position or tilt of the IOL may result in clinically significant refractive errors [3-5].

While refractive surprises following IOL implantation are most commonly attributed to biometric inaccuracies or capsular bag dynamics, structural alterations within the anterior segment remain an underreported cause [4,6,7]. Posterior synechiae, or adhesions of the posterior iris surface to the anterior lens capsule, can occur due to postoperative inflammation [8]. Localized synechiae may induce subtle biomechanical shifts, altering the ELP by tilting or anteriorly displacing the IOL complex.

We report an uncommon but underrecognized mechanism of a significant, symptomatic myopic shift secondary to localized posterior synechiae, resulting in anterior displacement and temporal tilt of an IOL following combined CLE and minimally invasive glaucoma surgery (MIGS). This refractive surprise was successfully reversed through targeted synechiolysis, restoring the ELP and resolving the patient’s symptoms.

## Case presentation

A 29-year-old man with underlying bilateral PIS and chronic angle-closure glaucoma, with a history of bilateral LPI performed at age 14 and bilateral ALPI two years earlier, presented with suboptimal intraocular pressure (IOP) control and disease progression despite maximal topical anti-glaucoma therapy. His left axial length (AL) was 21.95 mm. Preoperative optical biometry was performed using the IOLMaster 700 (Carl Zeiss Meditec, Jena, Germany). IOL power calculation was performed using the Barrett Toric Calculator (Alcon Laboratories, Inc., Fort Worth, TX, USA), with a target postoperative spherical equivalent of -0.50 D. To address the refractory IOP and anatomically narrow angles, the patient underwent a combined procedure in his left eye: CLE, goniosynechiolysis, implantation of a Clareon® Vivity® extended depth-of-focus (EDOF) toric IOL (Alcon Laboratories, Inc.), and insertion of three iStent infinite® (Glaukos Corporation, Aliso Viejo, CA, USA) devices under general anesthesia.

Intraoperatively, peripheral anterior synechiae (PAS) were noted predominantly in the nasal quadrant and were released with goniosynechiolysis until the trabecular meshwork became visible. Following successful angle opening and exposure of the trabecular meshwork within the nasal angle, three iStent infinite® (Glaukos Corporation) devices were implanted at the 7, 9, and 11 o'clock positions. Appropriate positioning within Schlemm's canal was confirmed by positive flow testing, which was observed over at least 5 clock hours. No residual PAS was observed at the completion of the procedure. The intraoperative course was otherwise uneventful. Postoperatively, the patient was treated with topical prednisolone acetate 1% (Pred Forte®; AbbVie Inc., Irvine, CA, USA) and levofloxacin 0.5% (Cravit®; Santen Pharmaceutical Co., Ltd., Osaka, Japan) every two hours as part of the routine postoperative regimen.

At the one-week postoperative review, the anterior chamber was deep, with 1+ cells and no evidence of fibrin, hypopyon, hyphema, or retained lens material. Topical prednisolone acetate 1% and levofloxacin 0.5% were tapered to four-hourly for two weeks, followed by four times daily for a further two weeks. No significant postoperative inflammatory reaction was documented during the early postoperative period.

Five weeks postoperatively, the patient presented with gradual-onset blurred vision in the left eye, binocular diplopia, asthenopia, and mild headaches. On examination, his best-corrected visual acuity (BCVA) was 6/36 in the left eye and 6/9 in the right eye. Refraction revealed a significant myopic surprise in the left eye of -2.50 DS/-0.25 DC × 65°, which significantly deviated from the intended target spherical equivalent of -0.50 D. His right eye refraction was +1.25 DS/-0.75 DC × 180°, indicating that his binocular diplopia and asthenopia were primarily driven by anisometropia-induced aniseikonia.

**Figure 1:**
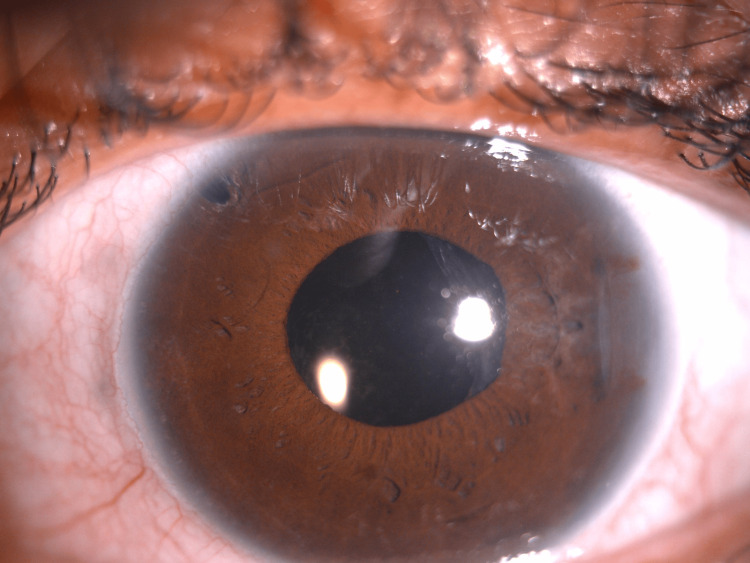
Subtle posterior synechiae on slit-lamp examination Anterior segment photo of the left eye showing a peripheral iridotomy at 10 o’clock and a relatively unremarkable anterior segment with subtle pupillary irregularity. Posterior synechiae were not clearly visible and were better appreciated on dynamic slit-lamp assessment.

**Figure 2:**
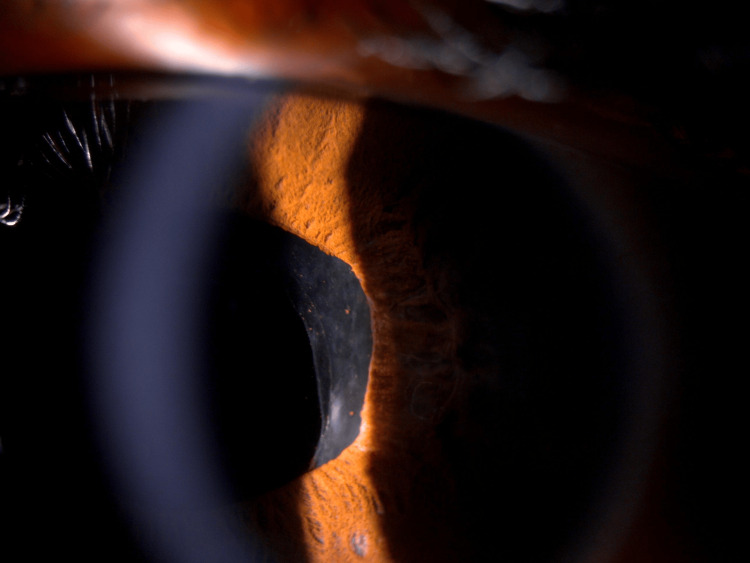
Posterior synechiae causing anterior displacement of the iris-lens diaphragm Angled slit-lamp photograph showing segmental posterior synechiae with focal adhesion between the iris and anterior lens capsule, contributing to anterior displacement of the iris-lens diaphragm.

Slit-lamp examination of the left anterior segment revealed a clear cornea and a deep anterior chamber with occasional inflammatory cells. A subtle pupillary irregularity was noted (Figure 1). Dynamic assessment of iris movement revealed localized posterior synechiae extending from 1 to 5 o’clock, causing focal adhesion between the iris and anterior capsule (Figure 2). While the IOL remained stable within the capsular bag, the localized synechial traction caused anterior displacement and temporal tilt of the IOL complex. The toric IOL axis was measured at 105°, compared with the intended implantation axis of 96°, representing a 9° deviation.

**Figure 3:**
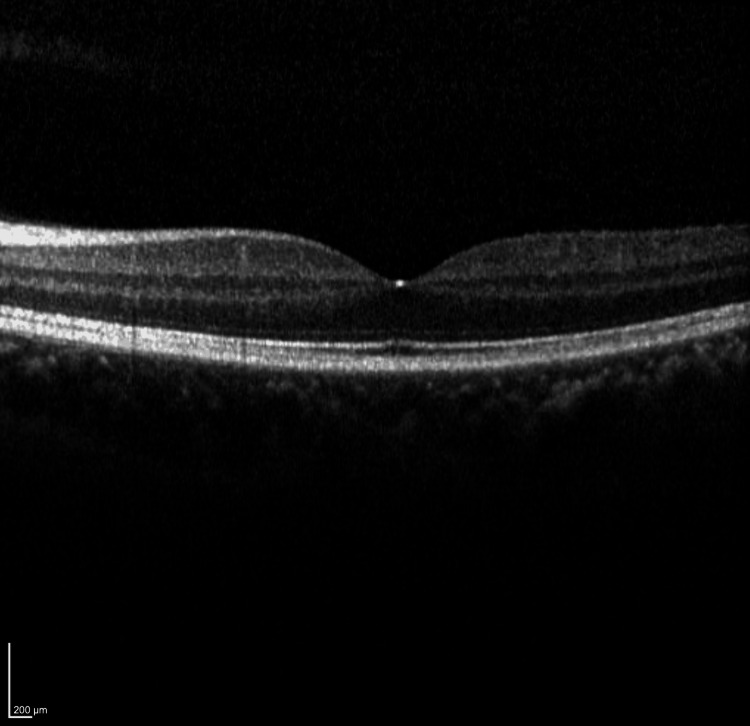
OCT of the left macula Macular OCT demonstrating preserved foveal contour without evidence of cystoid macular oedema or other significant macular pathology, supporting a non-retinal cause for the patient's postoperative visual symptoms. OCT: optical coherence tomography

Notably, the IOP in the left eye was well controlled at 12 mmHg without any anti-glaucoma eyedrops. Gonioscopy confirmed the optimal placement of all three iStent Infinite devices within the trabecular meshwork. Posterior segment examination demonstrated an advanced glaucomatous optic disc in his left eye, with a cup-to-disc ratio of 0.9 and a normal macula. Optical coherence tomography (OCT) of the macula confirmed a normal foveal contour without cystoid macular oedema (Figure 3).

Based on the clinical findings, a diagnosis of a myopic shift secondary to localized posterior synechiae altering the ELP was made. The patient subsequently underwent left-eye synechiolysis under local anesthesia. Following the injection of a cohesive ophthalmic viscoelastic device into the anterior chamber, the posterior synechiae were carefully released using a Kuglen hook (Bausch + Lomb Storz® Ophthalmic Instruments, St. Louis, MO, USA). The postoperative course was uneventful, with no adverse or unanticipated events following synechiolysis.

**Figure 4:**
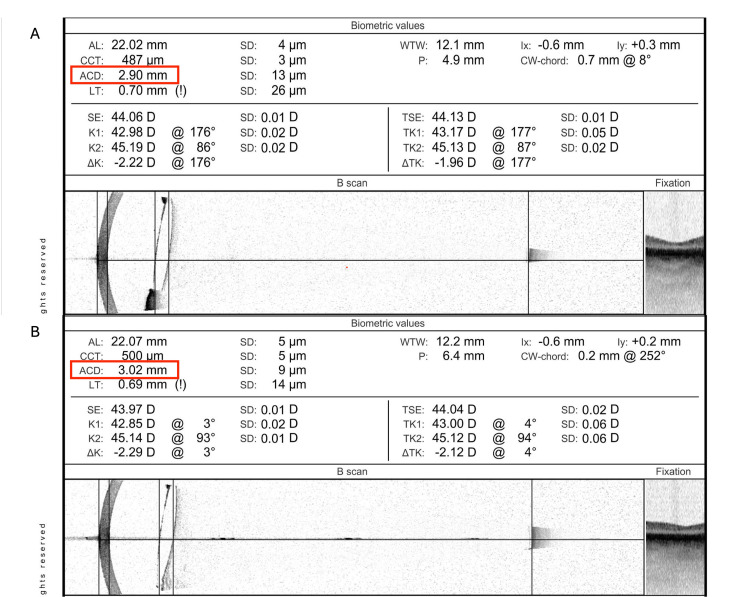
Comparison of ACD measurements before and after synechiolysis Biometry demonstrating ACD measurements before and after synechiolysis. (A) Biometry obtained at presentation showing a reduced ACD of 2.90 mm. (B) Repeat biometry following synechiolysis demonstrating deepening of the ACD to 3.02 mm, consistent with posterior repositioning of the IOL. ACD: anterior chamber depth; IOL: intraocular lens

Postoperatively, he reported complete resolution of his visual disturbances, binocular diplopia, and asthenopia. At one month following synechiolysis, BCVA improved to 6/15, while follow-up refraction demonstrated an improvement to -1.75 DS/-0.25 DC × 70°, substantially reducing the anisometropic difference and eliminating his symptoms. To objectively confirm the anatomical correction, repeat optical biometry using the IOLMaster 700 (Carl Zeiss Meditec) was performed, revealing a deepening of the anterior chamber depth (ACD) from 2.90 mm to 3.02 mm. This measurable posterior shift of the IOL confirmed the restoration of the ELP following the release of the synechial traction (Figure 4).

## Discussion

The management of PIS presents unique anatomical challenges. Because persistent angle closure in PIS is primarily driven by an anteriorly positioned and rotated ciliary body rather than pupillary block, standard LPIs are often insufficient. CLE has emerged as an increasingly utilized anatomical intervention. By removing the bulk of the crystalline lens, CLE increases the space in the anterior chamber, mitigates the effect of ciliary body anterior rotation, and helps relieve iridotrabecular contact [9]. Despite the anatomical benefits of CLE, predicting precise refractive outcomes in eyes with PIS and a short AL remains particularly challenging. In short eyes, such as the present case with an AL of 21.95 mm, a higher IOL power is required. Consequently, even minute deviations in the ELP translate into disproportionately large refractive surprises. Furthermore, the structurally crowded anterior segment, altered capsular dynamics, and unexpected postoperative ciliary body rotation in PIS eyes make ELP formulas less predictable [10].

This case illustrates an uncommon but clinically significant mechanism of postoperative refractive surprise due to anterior displacement of an IOL secondary to localized posterior synechiae. While most postoperative refractive surprises are attributed to biometric inaccuracies or generalized capsular contraction, the present case underscores an uncommon mechanical etiology [5,7,9,10]. Posterior synechiae typically result from intraocular inflammation, extensive manipulation during combined surgeries (such as CLE, goniosynechiolysis, and MIGS implantation), or complex anterior segment anatomy [8]. In addition, a relatively small or asymmetric capsulorrhexis may alter postoperative capsular bag dynamics through increased capsular contraction, potentially contributing to abnormal iris-capsular interactions [11].

In the present case, occasional anterior chamber inflammatory cells were observed at presentation. No significant postoperative inflammatory reaction was documented during follow-up. However, the persistence of occasional anterior chamber cells raises the possibility that low-grade postoperative inflammation may have contributed to the development of the localized posterior synechiae, although the exact mechanism cannot be established with certainty. The patient was compliant with the prescribed postoperative corticosteroid regimen, making medication non-adherence an unlikely contributor to synechiae formation.

When adhesions form between the iris and the anterior lens capsule, they exert asymmetrical mechanical traction on the capsular bag. This abnormal iris-lens interaction can tether the IOL complex, displacing it anteriorly and inducing a temporal tilt [12]. Interestingly, despite the clinically apparent temporal tilt, the induced refractive cylinder was minimal. This suggests that anterior displacement of the IOL, with alteration of the ELP, was the predominant mechanism underlying the refractive change, whereas the degree of tilt was likely subtle and contributed less to the overall refractive outcome. Because anterior displacement increases the effective optical power of the IOL, this mechanical shift manifested clinically as a sudden myopic surprise. Importantly, the synechiae were located posterior to the iris body rather than at the pupillary margin, making them less apparent on routine static examination.

Among the various factors influencing postoperative refractive outcomes, ELP is a key determinant of refractive accuracy, accounting for approximately 20%-40% of refractive prediction accuracy in cataract surgery [4,13]. ELP describes the axial position of the IOL within the eye and represents the balance of forces acting on the capsular bag, including capsular contraction and fibrosis, which may result in anterior or posterior displacement of the IOL [7,13]. Anterior displacement increases the effective optical power of the IOL, resulting in a myopic shift, whereas posterior displacement results in a hyperopic shift [4].

In this case, the optical consequences of this displacement were twofold. First, the implantation of a premium EDOF IOL likely exacerbated the patient’s subjective visual disturbances, as complex multifocal and EDOF optics are exquisitely sensitive to tilt, decentration, and axial misalignment [14]. Second, the unilateral myopic shift (-2.50 D) created a profound anisometropia compared to the fellow eye. Sudden-onset anisometropia exceeding 1.50 to 2.00 D disrupts binocular fusion, leading to clinically significant aniseikonia, asthenopia, and binocular diplopia [15]. While conservative management of anisometropia-induced diplopia typically includes prismatic spectacle correction, contact lenses, or contralateral refractive surgery, the definitive management in this context necessitated addressing the root anatomical cause.

The actual postoperative position of the IOL differs from the predicted ELP and can be assessed clinically using ACD measurements [13]. In this case, the observed increase in ACD from 2.90 mm to 3.02 mm following synechiolysis provides objective evidence supporting this mechanism. Although the increase in ACD supports posterior repositioning of the IOL, the magnitude of the measured change alone may not fully account for the observed refractive improvement. Additional optical factors, including subtle IOL tilt, decentration, or asymmetric capsular bag forces related to the localized posterior synechiae, may also have contributed. Because postoperative anterior segment OCT and ultrasound biomicroscopy (UBM) were not available, the relative contribution of these factors could not be quantified. Alternative explanations, including corneal and accommodative factors, were considered less likely given the patient's pseudophakic status and the objective increase in ACD following synechiolysis.

Although ELP is a theoretical construct used in IOL power calculations, it may not accurately reflect the true postoperative position of the IOL, as anatomical changes and surgical factors can alter its final location [13,16]. Discrepancies between estimated and actual IOL position have been reported and may contribute to unexpected refractive outcomes [13,16]. The residual myopia observed following synechiolysis may also reflect the inherent challenges of refractive prediction in eyes with PIS and short AL. In addition to the mechanical effects of the localized posterior synechiae, limitations in ELP prediction and other postoperative anatomical changes may have contributed to the residual refractive error.

This case highlights the importance of careful anterior segment evaluation in patients with postoperative refractive surprise. Mechanical causes, including posterior synechiae, should be considered in patients presenting with new-onset myopic shift following IOL implantation, particularly in those with complex anterior segment conditions. Recognition of this mechanism is crucial, as it represents a reversible cause of refractive error when treated appropriately.

This report has several limitations. Although the initial myopic shift was clearly documented, comprehensive postoperative imaging, such as anterior segment OCT or UBM, was not available to objectively quantify the exact vector of IOL tilt and positional restoration. Nevertheless, the measurable increase in ACD and complete resolution of visual symptoms support the proposed mechanism.

## Conclusions

Localized posterior synechiae can mechanically tether the capsular bag, resulting in anterior displacement and tilt of an IOL. This leads to a clinically significant myopic shift, anisometropia, and binocular diplopia, which can be particularly debilitating in eyes with EDOF lenses. This case highlights an uncommon but potentially reversible cause of postoperative refractive surprise that may be easily overlooked when the IOL appears well centered. Meticulous dynamic slit-lamp evaluation under pupillary dilation, paired with careful ACD tracking, is essential for an accurate diagnosis in high-risk eyes with complex anterior segment conditions. Synechiolysis serves as an effective, targeted intervention to restore the ELP while improving visual function.
